# Knee osteoarthritis and adverse health outcomes: an umbrella review of meta-analyses of observational studies

**DOI:** 10.1007/s40520-022-02289-4

**Published:** 2022-11-04

**Authors:** Nicola Veronese, Germain Honvo, Olivier Bruyère, René Rizzoli, Mario Barbagallo, Stefania Maggi, Lee Smith, Shaun Sabico, Nasser Al-Daghri, Cyrus Cooper, Francesco Pegreffi, Jean-Yves Reginster

**Affiliations:** 1 Geriatric Unit, Department of Internal Medicine and Geriatrics, University of Palermo, Via del Vespro, 141, 90127 Palermo, Italy; 2 Chair for Biomarkers of Chronic Diseases, Biochemistry Department, College of Science, King Saud University, Riyadh, Saudi Arabia; 3 World Health Organization (WHO) Collaborating Center for Public Health Aspects of Musculoskeletal Health and Aging, University of Liège, Liège, Belgium; 4 Division of Public Health, Epidemiology and Health Economics, University of Liège, Liège, Belgium; 5 Division of Bone Diseases, Geneva University Hospitals and Faculty of Medicine, Geneva, Switzerland; 6 CNR-Neuroscience Institute, Aging Branch, Padua, Italy; 7 Centre for Health Performance and Wellbeing, Anglia Ruskin University, Cambridge, UK; 8 MRC Lifecourse Epidemiology Unit, Southampton General Hospital, University of Southampton, Southampton, UK; 9 Department for Life Quality Studies, University of Bologna, Bologna, Italy

**Keywords:** Knee osteoarthritis, Cardiovascular disease, Falls, Mortality, Meta-analysis, Umbrella review

## Abstract

**Background:**

Knee osteoarthritis (OA) is a common condition, associated with a high rate of disability and poor quality of life. Despite the importance of such evidence in public health, no umbrella review (i.e., a review of other systematic reviews and meta-analyses) has systematically assessed evidence on association between knee OA and adverse health outcomes.

**Aims:**

To map and grade all health outcomes associated with knee OA using an umbrella review approach.

**Methods:**

The search was made across several databases up to 22 April 2022. We used an umbrella review of systematic reviews with meta-analyses of observational studies assessing the effect sizes, based on random effect summary, 95% prediction intervals, heterogeneity, small study effects, and excess significance bias. The evidence was then graded from convincing (class I) to weak (class IV).

**Results:**

Among 3,847 studies initially considered, five meta-analyses were included for a total of five different outcomes. Three adverse outcomes were significantly associated with knee OA (i.e., cardiovascular mortality, falls, and subclinical atherosclerosis). The presence of knee OA was associated with a significantly higher risk of cardiovascular mortality (odds ratio, OR = 1.17; 95%CI, confidence intervals: 1.02–1.34), falls (RR = 1.34; 95%CI: 1.10–1.64), and conditions associated with subclinical atherosclerosis (OR = 1.43; 95%CI: 1.003–2.05). The certainty of each of this evidence was weak.

**Conclusions:**

Our umbrella review suggests that knee OA can be considered as putative risk factor for some medical conditions, including cardiovascular diseases and falls, however, it is important to note that the evidence is affected by potential biases.

**Supplementary Information:**

The online version contains supplementary material available at 10.1007/s40520-022-02289-4.

## Introduction

Osteoarthritis (OA) is a common condition, characterized by joint pain and stiffness with relevant consequences on functional status, significantly restricting daily activities, and often resulting in a reduction in quality of life [1, 2]. Knee OA is the most common site of OA [3]. It is estimated that symptomatic forms may affect more than 250 million people worldwide [3]. Moreover, due to extensive structural abnormalities in cellular tissue of cartilage, subchondral bone, synovium, capsule, and ligaments leading to pain upon movement and functional limitation, knee OA is ranked among the most common causes of global disability in terms of disability-adjusted life years (DALY) [4–7].

Increasing literature has reported that knee OA could be considered as a potential risk factor for other non-communicable diseases [8] such as cardiovascular [9] and metabolic conditions [10, 11], as well as mortality [8, 12]. Despite the importance of such evidence in public health, to the best of our knowledge, no umbrella review (i.e., a review of other systematic reviews and meta-analyses) has systematically assessed evidence on association between knee OA and related adverse health outcomes (i.e., non-communicable diseases or mortality).

Therefore, the present study addresses the question: what are the adverse health outcomes (i.e., non-communicable diseases or mortality) for which knee OA is a potential risk factor, based on current evidence from systematic reviews of observational studies? The purpose is to highlight the impact of knee OA on patients and health systems, beyond its direct consequences on functional status and quality of life of patients.

## Methods

### Protocol and registration

This study was conducted following the recommendations of the Joanna Briggs Institute (JBI) Collaboration for conducting umbrella reviews [13] and those reported in the Cochrane Handbook for Systematic Literature Reviews to carry out the screening and selection of studies [14]. The protocol is fully available at https://osf.io/rb9qt/. The findings of the umbrella review were reported according to the updated 2020 Preferred Reporting Items for Systematic Review and Meta-Analysis (PRISMA) guidelines [15].

### Information sources and search strategies

For the conduct of this umbrella review, several relevant bibliographic databases were comprehensively searched, including Medline (via Ovid), Embase, Scopus, and CINAHL (Cumulative Index to Nursing and Allied Health Literature) each from inception up to 22nd April 2022.

To guide the identification of adequate key words for building search strategies to search these bibliographic databases, the research question was framed into PICOS (Participants, Intervention, Comparison, Outcome, Study type) format, knowing that there is no intervention in an umbrella review of observational studies. The research question formulated into PICOS format is as follows:

In people with knee OA (P), compared to those without knee OA (C), what are health outcomes (i.e., non-communicable diseases or mortality) associated with knee OA condition (O), based on evidence from systematic reviews with meta-analyses of observational studies(S)?

We built detailed and highly sensitive search strategies combining search terms (free vocabulary words and controlled vocabulary terms) from the main PICOS elements, tailored to the syntax of each of the databases considered for this umbrella review, as shown in Supplementary Table 1.

### Eligibility criteria

In this umbrella review, we included systematic reviews with formal meta-analyses of observational studies that investigated the relationship between knee OA and any adverse health outcome. Specific inclusion criteria included the following: (1) systematic reviews containing sufficient data for a meta-analysis that assessed knee OA and ascertained health outcomes using self-report (e.g., depression questionnaires), observed (e.g., clinical diagnoses), or objective (e.g., biomarkers and mortality) criteria; and (2) meta-analyses of case–control or cohort studies that investigated the association of knee OA with any adverse health-related outcome (e.g., cardiovascular disease, cancer, death, obesity/overweight, mental illness, diabetes, and metabolic diseases, etc.). Studies had to report these outcomes as odds ratio (OR), relative risk (RR), hazard ratio (HR), or mean and standard deviation or standardized mean differences (for continuous data). We extracted, if available, the estimate adjusted for the highest number of potential confounders. Meta-analyses of cross-sectional studies were excluded from this umbrella review, because in cross-sectional studies, the temporal link between the outcome and the exposure cannot be determined since both are examined at the same time (temporal bias).

### Study selection

We followed the recommendations in the Cochrane Handbook for Systematic Reviews to select studies that were finally included in this review [14]. The selections were independently carried out by two review authors (SS, FP), with consensus meetings to discuss the studies for which divergent selection decision were made by the two review authors. A third member of the review team (NV) was involved, if necessary. The studies selection process involved, first, a selection based on title and/or abstracts, then a selection of studies retrieved from this first step based on the full-text manuscripts. The Covidence online software (https://www.covidence.org/) was used to manage the entire study selection process.

### Data collection and data items

Items collected from the retrieved full-text articles were: information for identification of the included meta-analyses (e.g., first author name and affiliation, year of publication, journal name, title of the manuscript), data on the characteristics of the population considered, for individual observational studies (e.g., sample size, mean age, location, gender, etc.), as well as data on diagnostic criteria for knee OA (e.g., radiological, clinical, medical records etc.), the study design, the number of cases (i.e., incident events) and controls (non-events), the number of exposed and unexposed participants), for each individual observational study included in each retrieved systematic review, and health outcomes. These data were collected using a standardized data extraction form. The form was pre-tested by the review authors leading on data extraction. The data extraction was carried out by one review author (SB) and systematically cross checked by a second review author (NV). Errors found in extraction by the second review author were corrected during an online consensus meeting by both authors.

### Assessment of risk of bias

One author (FP) independently rated the methodological quality of the included systematic reviews using “A MeaSurement Tool to Assess systematic Reviews 2 (AMSTAR 2)”, which ranks the quality of a meta-analysis in 1 of 4 categories ranging from “critically low” to “high” according to 16 predefined items [16], with another author (NV) cross-checking these assessments.

### Data synthesis

The data analysis was conducted using STATA 14.0. For each meta-analysis, we estimated the common effect size and its 95%CI (confidence interval) through random-effects models [17]. We also estimated the prediction interval and its 95% CI, which further accounts for between-study effects and estimates the certainty of the association if a new study addresses that same association [18]. Between-study heterogeneity was estimated with the *I*^2^ statistics; values of 50% or greater are indicative of high heterogeneity, while values above 75% suggest very high heterogeneity [19].

In addition, we evaluated the evidence of small study effects (i.e., whether small studies would have inflated effect sizes compared to larger ones) [20]. To this end, we used the regression asymmetry test developed by Egger and co-workers [21]. A *p* value of less than 0.10 was considered as indicative of small-study effects, while a *p*-value < 0.05 of publication bias. Moreover, we considered if the largest study in terms of participants included was statistically significant or not. Finally, we applied Ioannidis's excess of significance test to evaluate whether there was an excess of studies reporting statistically significant results [22].

### Grading the evidence

Using the tests described in the previous paragraph, we used the credibility assessment criteria, based on established tools for observational evidence [23]. We classified the evidence available from meta-analyses of observational studies with nominally statistically significant summary results (*p* < 0.05) into four different categories (Classes I, II, III, and IV). Associations were considered to be convincing (Class I) if they had (1) a statistical significance of *p* value of less than 10^–6^, (2) included more than 1000 cases (or more than 20,000 participants for continuous outcomes), (3) had the largest component study reporting a significant result (*p* < 0.05), (4) had a 95% prediction interval that excluded the null, (5) did not have large heterogeneity (*I*^2^ < 50%), and (6) showed no evidence of small study effects (*p* > 0.10) or of excess significance bias (*p* > 0.10). Highly suggestive (Class II) evidence was assigned to associations that (1) reported a significance of *p* values of less than 0.001, (2) included more than 1000 cases (or more than 20,000 participants for continuous outcomes), and (3) had the largest component study reporting a statistically significant result (*p* < 0.05). Suggestive (Class III) evidence was assigned to associations that reported a significance of a *p* value of less than 0.01 with more than 1000 cases (or more than 20,000 participants for continuous outcomes). Weak (Class IV) evidence was assigned to the remaining significant associations with a *p* value of less than 0.05.

Due to the inherent limitations of case–control studies in examining temporal associations, we plan to provide the classification of evidence for Class I and Class II including only cohort/prospective studies. Outcomes in Class I or Class II were assessed also in terms of possible risk of bias evaluated with the AMSTAR-2. However, since no outcome reached this level of evidence, these analyses were not reported.

### Ethical issues

This study did not involve patients or any human or animal material, and therefore does not imply any ethical issue.

## Results

### Literature search

**Fig. 1:**
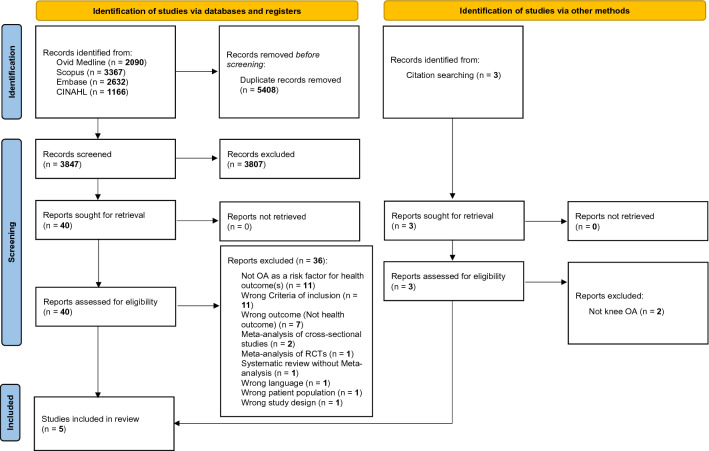
PRISMA flow-chart

As shown in Fig. 1, among 3,847 papers initially considered from databases search, we assessed 40 full texts. After excluding 36 works (list reported in Supplementary Table 2), mainly based on the fact that knee OA was not considered as risk factor for other health outcomes or that wrong criteria for inclusion (e.g., mixed forms of OA instead of knee OA) were used, four meta-analyses were included. One additional meta-analysis was identified through manual search of references of the studies included from databases search, so that we finally included five meta-analyses in this umbrella review [24–28].

### Main findings of the umbrella review

As shown in Table 1, the five meta-analyses included a median of four studies (range: 3–7) for outcome with a median of 11,071 participants, followed-up for a median of 8 years (range: 7–12). The median of the mean ages from the included studies was 65.1 years, with a median percentage of females of 67.2%. The studies included different methods for the diagnosis of knee OA, including clinical, radiological or mixed information (Table 1).

**Table 1:** Main findings of the meta-analyses included

Author, year	Number of studies	Sample size	Type of studies included	Mean age	% females	Diagnosis of knee OA	Outcome	Mean follow-up time (years)	Type of metric	Effect size	*P* value	I^2^	Small study effects	Publication bias	Excess significance bias	Largest study significant	Prediction intervals	Level of evidence
Veronese, 2016; Leyland, 2021	7	10,320	Cohort	62.9	69.2	Clinical + Radiological (4 studies); only radiological (3 studies)	All-cause mortality	9	HR	1.18 (0.95–1.46)	0.13	63.9	No	No	No	No	0.64–1.84	NS
Macedo,2022; Wang, 2016	4	44,095	Cohort	65.1	63	Clinical (2 studies); radiological (2 studies)	CV Mortality	12	OR	1.17 (1.02–1.34)	0.03	84.3	Yes	No	Yes	Yes	0.68–2.21	IV
Macedo,2022; Wang, 2016	3	11,071	Cohort	65.9	67.2	Clinical + Radiological (2 studies); radiological (1 study)	CVD	8	HR	1.09 (0.97–1.24)	0.16	31.2	No	No	Not possible	No	0.80–2.15	NS
Deng, 2020	4	10,985	Cohort	69.4	79.9	Radiological (3 studies); Clinical + Radiological (1 study)	Falls	7	RR	1.34 (1.10–1.64)	0.004	75.9	No	No	No	No	0.84–1.54	IV
Macedo, 2022	4	11,083	Cohort; case–control	64.3	58	Radiological (3 studies); clinical (1 study)	Subclinical Atheroclerosis	8	OR	1.43 (1.003–2.05)	0.043	71.5	Yes	Yes	Not possible	Yes	0.85–2.84	IV

*OA* Osteo Arthritis, *CV* Cardio Vascular, *CVD* Cardio Vascular Disease, *HR* Hazard Ratio, *OR* Odds Ratio, *RR* Relative Risk, *P value* Probability value, *I*^*2*^ Index of Heterogeneity, *NS* Non Significant

Among the five outcomes identified from the meta-analyses, the majority considered cardiovascular conditions (incident cardiovascular disease, cardiovascular mortality, and conditions associated with subclinical atherosclerosis), together with all-cause mortality and falls. Three adverse outcomes were significantly associated with knee OA (i.e., cardiovascular mortality, falls, and subclinical atherosclerosis), with *p* values < 0.05. For all the outcomes, except incident cardiovascular diseases, the common effect size was associated with a high heterogeneity (defined as an I^2^ ≥ 50%). Small study effect was present in two outcomes (cardiovascular mortality and subclinical atherosclerosis), while only subclinical atherosclerosis was associated with the presence of a possible publication bias. Excess significance bias was present for the data regarding cardiovascular mortality. Finally, the largest study in terms of participants showed statistically significant results only in two outcomes (cardiovascular mortality and subclinical atherosclerosis), while prediction intervals including the null effect value (i.e., 1.0 for RR or OR) was found for all the outcomes reported (Table 1).

Based on the abovementioned criteria, for all the outcomes with statistically significant results, the certainty of evidence was rated as weak. In summary, the presence of knee OA was associated with a significantly higher odds or risk of cardiovascular mortality (OR = 1.17; 95%CI: 1.02–1.34), falls (RR = 1.34; 95%CI: 1.10–1.64), and conditions associated with subclinical atherosclerosis (OR = 1.43; 95%CI: 1.003–2.05) (Table 1), but the certainty of each of this evidence was weak.

### Assessment of risk of bias

Using the criteria suggested by the AMSTAR-2, four meta-analyses were rated as critically low and one low, in terms of methodological quality (Supplementary Table 3). The most common reasons of potential bias were “not clear declaration of the PICO question” (question 1), “protocol not published before the work” (question 2), and “missing information regarding the possible role of publication bias in meta-analyses” (question 15).

## Discussion

In this umbrella review including five systematic reviews with meta-analysis, we found that the presence of knee OA significantly increased the risk of some medical conditions, particularly of cardiovascular nature, and falls. However, it should be noted that these findings were supported by a weak level of evidence; the attempt to identify how knee joint disease influences morbidity and mortality is of paramount importance for public health.

The association between knee OA and cardiovascular diseases, in our case in subclinical forms and cardiovascular mortality, may be explained by several reasons. First, knee OA and cardiovascular diseases have common risk factors such as age and obesity. Moreover, knee OA can increase the risk of traditional risk factors for cardiovascular diseases, i.e., hypercholesterolemia [29], hypertension [30], aortic calcification [31], lumbar atherosclerotic calcification [32] or diabetes [33, 34]. Of importance, a study with a 3 year follow-up reported a strong association between hypertension and impaired glucose tolerance with knee OA occurrence [35]. Furthermore, in knee OA patients with hypertension and type 2 diabetes mellitus, greater bone loss in the subchondral plate has been reported, compared with subjects with healthy knee [36]. Progressive structural changes in joint morphology results in altered anatomy and consequently in range of motion reduction leading to forced physical inactivity [37]. The latter aspect is one of the most recognized risk factors for both cardiovascular disease and mortality [37]. Second, it was proposed that people with knee OA may use a greater number of anti-inflammatory drugs, some of them leading to cardiovascular impairment [38]. Finally, it is largely known that knee OA may promote sedentary behaviors, mood disorders, and frailty/disability, that can further increase the risk of cardiovascular risk [37]. Other literature has also proposed that changes in extracellular matrix remodeling or altered Wnt signaling transduction, that are present in knee OA, can contribute to subclinical and fatal cardiovascular diseases [39, 40]. All these findings suggest that, in people with knee OA, it is important not only to early detect cardiovascular diseases in subclinical forms, but also to strictly monitor those with a high cardiovascular risk.

Another significant result of our umbrella review is that knee OA significantly increased the risk of falls. In addition to the reasons cited before and that can apply to justify this epidemiological association, it is largely known that people with knee OA are prone to postural instability that can increase the risk of falls [41]. Functional impairment of the knee joint affected by osteoarthritis and loss of muscle strength lead to a significant increase in the number of falls. Compensatory strategies of patients with knee OA in response to a backward slip perturbation compared with healthy older adults must be considered. In a recent paper comparing healthy older individuals with those who have knee OA, it was observed that those with knee OA have a higher risk of falling in response to a backward slip perturbation [42]. Moreover, an increase in the number of joints with symptomatic OA of the lower extremities increases the risk of falling [43].

The findings of our study must be interpreted within its limitations. First, the number of meta-analyses included satisfying the selection criteria was limited to only five works with five outcomes, overall indicating the need of more research. Second, the significant associations found between knee OA and health outcomes were affected by some potential biases that ultimately lead to a weak strength of evidence for all these associations. In this sense, we believe that further meta-analyses with larger studies can help overcome these shortcomings. Third, we found a high heterogeneity (as I^2^ more than 50%) that probably reflects some clinical differences including different definitions of knee OA, follow-up durations, definition of outcomes, and potentially others. Finally, according to the AMSTAR 2 evaluation, the meta-analyses included were rated as critically low or low quality, possibly introducing other biases.

In conclusion, our umbrella review suggests that knee OA can be considered a putative risk factor for some medical conditions, including cardiovascular diseases and falls; however, the evidence was affected by several potential biases and likely mediated by confounders. Since knee OA is the most common musculoskeletal disease, further research is urgently needed for better understanding its epidemiological importance as risk factor for medical conditions.

## Supplementary Information

Below is the link to the electronic supplementary material.

Supplementary file1 (DOCX 25 KB)
