# Supplementary material for: Knee osteoarthritis and adverse health outcomes: an umbrella review of meta-analyses of observational studies
Source: Aging Clin Exp Res. 2022 Nov 4;35(2):245–52. doi: 10.1007/s40520-022-02289-4 (PMC9895027; doi:10.1007/s40520-022-02289-4)
Supplement: Supplementary file 1 — Supplementary file1 (DOCX 25 KB) [file 40520_2022_2289_MOESM1_ESM.docx]

**Supplementary Table 1. Search strategy**

Database: **Ovid MEDLINE(R) ALL** <1946 to April 22, 2022>

Search Strategy:

--------------------------------------------------------------------------------

1 Osteoarthritis, Knee/

2 (knee adj2 (osteoarthr* or arthros* or "joint failure*" or "joint degenerat*" or "osteo-arthritis" or "OA")).ti,ab,kw.

3 (gonarthros* or gonarthriti*).ti,ab,kw.

**4 1 or 2 or 3**

5 meta-analysis/ or "systematic review"/

6 meta-analysis as topic/ or Systematic Reviews as Topic/

7 Meta-Analysis.pt.

8 Systematic Review.pt.

9 ("meta-analysis" or metaanalysis or "data pooling*" or "evidence synthesis" or "systematic review").ti,ab,kw.

**10 5 or 6 or 7 or 8 or 9**

**11 4 and 10**

***************************

Database: **SCOPUS**

Search Strategy:

--------------------------------------------------------------------------------

1 TITLE-ABS-KEY (knee W/2 (osteoarthr* OR arthros* OR "joint failure*" OR "joint degenerat*" OR "osteo-arthritis" OR "OA"))

2 TITLE-ABS-KEY (gonarthros* OR gonarthriti*)

**3 #1 OR #2**

4 TITLE-ABS-KEY ("meta-analysis" OR metaanalysis OR "data pooling*" or "evidence synthesis" or "systematic review")

**5 #3 AND #4**

**Combined form**

( ( TITLE-ABS-KEY ( knee W/2 ( osteoarthr* OR arthros* OR "joint failure*" OR "joint degenerat*" OR "osteo-arthritis" OR "OA" ) ) ) OR ( TITLE-ABS-KEY ( gonarthros* OR gonarthriti* ) ) ) AND ( TITLE-ABS-KEY ( "meta-analysis" OR metaanalysis OR "data pooling*" OR "evidence synthesis" OR "systematic review" ) )

Database: **Embase**

Search Strategy:

--------------------------------------------------------------------------------

1 'knee osteoarthritis'/de

2 (knee NEAR/3 (osteoarthr* OR arthros* OR "joint failure*" OR "joint degenerat*" OR "osteo-arthritis" OR "OA")):ti,ab,kw

3 (gonarthros* OR gonarthriti*):ti,ab,kw

**4 #1 OR #2 OR #3**

5 'meta analysis'/de OR 'systematic review'/de

6 'meta analysis (topic)'/de OR 'systematic review (topic)'/de

7 ("meta-analysis" OR metaanalysis OR "data pooling*" OR "evidence synthesis" OR "systematic review"):ti,ab,kw

8 **#5 OR #6 OR #7**

**9 #4 AND #8**

**10 #9 AND ('article'/it OR 'article in press'/it OR 'review'/it)**

Database: **CINAHL**

Search Strategy:

--------------------------------------------------------------------------------

1 (MH "Osteoarthritis, Knee")

2 AB (knee N2 (osteoarthr* OR arthros* OR "joint failure*" OR "joint degenerat*" OR "osteo-arthritis" OR "OA"))

3 AB (gonarthros* OR gonarthriti*)

**4 S1 OR S2 OR S3**

5 (MH "Meta Analysis") OR (MH "Systematic Review")

6 AB ("meta-analysis" OR metaanalysis OR "data pooling*" OR "evidence synthesis" OR "systematic review")

**7 S5 OR S6**

**8 S4 AND S7**

Limit to: Source Types “Academic Journals”

**Supplementary Table 2. List of excluded references, with reason.**

| **Study** | **Title** | **Reason of exclusion** |
| --- | --- | --- |
| **Baudart 2017** | Association between osteoarthritis and dyslipidaemia: a systematic literature review and meta-analysis. | Wrong Criteria |
| **Betzler 2022** | The Prevalence of Coexisting Lumbar Spondylosis and Knee Osteoarthritis: A Systematic Review and Meta-Analysis. | Wrong Criteria |
| **Dainese 2022** | Association between knee inflammation and knee pain in patients with knee osteoarthritis: a systematic review. | Wrong outcome (Not health outcome) |
| **Deasy 2016** | Hip Strength Deficits in People With Symptomatic Knee Osteoarthritis: A Systematic Review With Meta-analysis. | Wrong outcome (Not health outcome) |
| **Eitner 2021** | Diabetes - Osteoarthritis and joint pain | Systematic review without Meta-analysis |
| **Fingleton 2015** | Pain sensitization in people with knee osteoarthritis: a systematic review and meta-analysis. | Wrong Criteria |
| **French 2017** | Prevalence of neuropathic pain in knee or hip osteoarthritis: A systematic review and meta-analysis. | Meta-analysis of cross-sectional studies |
| **Hart 2019** | Is quality of life reduced in people with patellofemoral osteoarthritis and does it improve with treatment? A systematic review, meta-analysis and regression. | Wrong Criteria |
| **Hatfield 2016** | Clinical Tests of Standing Balance in the Knee Osteoarthritis Population: Systematic Review and Meta-analysis. | Wrong Criteria |
| **Hoch 2011** | Serum cartilage oligomeric matrix protein (sCOMP) is elevated in patients with knee osteoarthritis: a systematic review and meta-analysis. | Wrong outcome (Not health outcome) |
| **Hussain 2020** | Vascular Pathology and Osteoarthritis: A Systematic Review. | Not OA as a risk factor for health outcome(s) |
| **Iijima 2018** | Biomechanical characteristics of stair ambulation in patients with knee OA: A systematic review with meta-analysis toward a better definition of clinical hallmarks. | Wrong outcome (Not health outcome) |
| **Klit 2011** | [Arthrosis and arthroplastic surgery influence patients' sex life, depression, and socioeconomy]. | Wrong language (Article not in English) |
| **Lee 2012** | Obesity and knee osteoarthritis. | Wrong Criteria |
| **Lo 2022** | Association between hypertension and osteoarthritis: A systematic review and meta-analysis of observational studies | Not OA as a risk factor for health outcome(s) |
| **Manlapaz 2019** | Risk Factors for Falls in Adults with Knee Osteoarthritis: A Systematic Review. | Not OA as a risk factor for health outcome(s) |
| **Master 2021** | A Narrative Review on Measurement Properties of Fixed-distance Walk Tests Up to 40 Meters for Adults With Knee Osteoarthritis. | Wrong Criteria |
| **Mayburd 2019** | Increased lifespan, decreased mortality, and delayed cognitive decline in osteoarthritis. | Wrong study design (Not a systematic review) |
| **Milaras 2021** | Association of Matrix Metalloproteinase (MMP) Gene Polymorphisms With Knee Osteoarthritis: A Review of the Literature. | Not OA as a risk factor for health outcome(s) |
| **Mills 2013** | Biomechanical deviations during level walking associated with knee osteoarthritis: a systematic review and meta-analysis. | Wrong Criteria |
| **Nie 2020** | Metabolic syndrome and the incidence of knee osteoarthritis: A meta-analysis of prospective cohort studies. | Not OA as a risk factor for health outcome(s) |
| **Pedroso 2019** | Fatty infiltration in the thigh muscles in knee osteoarthritis: a systematic review and meta-analysis. | Wrong Criteria |
| **Pietrosimone 2011** | Voluntary quadriceps activation deficits in patients with tibiofemoral osteoarthritis: a meta-analysis. | Wrong outcome (Not health outcome) |
| **Scarvell 2018** | Kinematics of knees with osteoarthritis show reduced lateral femoral roll-back and maintain an adducted position. A systematic review of research using medical imaging. | Wrong outcome (Not health outcome) |
| **Schulz 2020** | Are we missing the target? Are we aiming too low? What are the aerobic exercise prescriptions and their effects on markers of cardiovascular health and systemic inflammation in patients with knee osteoarthritis? A systematic review and meta-analysis. | Meta-analysis of RCTs |
| **Sonoo 2019** | Altered sagittal plane kinematics and kinetics during sit-to-stand in individuals with knee osteoarthritis: A systematic review and meta-analysis. | Not OA as a risk factor for health outcome(s) |
| **Tang 2018** | Association of osteoarthritis and circulating adiponectin levels: A systematic review and meta-analysis | Not OA as a risk factor for health outcome(s) |
| **Tayfur 2022** | Neuromuscular joint function in knee osteoarthritis: a systematic review and meta-analysis. | Wrong Criteria |
| **vanderWaal 2005** | The impact of non-traumatic hip and knee disorders on health-related quality of life as measured with the SF-36 or SF-12. A systematic review. | Wrong patient population |
| **Wang 2019** | Association between chondrocalcinosis and osteoarthritis: A systematic review and meta-analysis. | Not OA as a risk factor for health outcome(s) |
| **Webster 2022** | Anterior Cruciate Ligament Injury and Knee Osteoarthritis: An Umbrella Systematic Review and Meta-analysis. | Not OA as a risk factor for health outcome(s) |
| **Xie 2021** | Metabolic syndrome, hypertension, and hyperglycemia were positively associated with knee osteoarthritis, while dyslipidemia showed no association with knee osteoarthritis. | Wrong Criteria |
| **Xiong 2020** | Dyslipidemia Might Be Associated with an Increased Risk of Osteoarthritis. | Not OA as a risk factor for health outcome(s) |
| **Young 2022** | Prevalence of multimorbid degenerative lumbar spinal stenosis with knee or hip osteoarthritis: a systematic review and meta-analysis. | Meta-analysis of cross-sectional studies |
| **Zhang 2017** | Association between hypertension and risk of knee osteoarthritis: A meta-analysis of observational studies. | Not OA as a risk factor for health outcome(s) Germain Honvo (2022-06-21 19:28:08)(Select): “Not OA as risk factor” => Exclude |
| **Zolio 2021** | Systematic review and meta-analysis of the prevalence of neuropathic-like pain and/or pain sensitization in people with knee and hip osteoarthritis. | Wrong outcome (Not health outcome) |

**Supplementary Table 3. AMSTAR 2 quality assessment of meta-analyses included**

| **Author, Year** | **1** | **2** | **3** | **4** | **5** | **6** | **7** | **8** | **9** | **10** | **11** | **12** | **13** | **14** | **15** | **16** | **Overall rating** |
| --- | --- | --- | --- | --- | --- | --- | --- | --- | --- | --- | --- | --- | --- | --- | --- | --- | --- |
| **Deng, 21** | no | no | yes | partial yes | yes | no | Yes | yes | no | no | no | no | yes | no | no | yes | **Critically low** |
| **Leyland, 21** | no | no | yes | yes | no | no | Yes | partial yes | no | yes | no | no | no | no | no | yes | **Critically low** |
| **Macedo, 22** | no | no | yes | partial yes | no | no | Yes | partial yes | no | yes | no | no | no | yes | no | yes | **Critically low** |
| **Veronese, 16** | no | no | yes | yes | yes | yes | Yes | yes | yes | yes | yes | no | no | yes | no | yes | **Low** |

Notes:

1. Did the research questions and inclusion criteria for the review include the components of **PICO** (Population, Intervention, Comparator group, Outcome)? YES/NO. For yes, must have all four.

2. Did the report of the review contain an **explicit statement that the review methods were established prior to the conduct of the review** and did the report justify any significant deviations from the protocol? YES, PARTIAL YES, NO. For Partial YES: the authors state that they had a written protocol or guide that included ALL the following (review question(s), a search strategy, inclusion/exclusion criteria, a risk of bias assessment). For YES: as for partial yes, plus the protocol should be registered and should also have specified: a meta-analysis/synthesis plan, if appropriate, and a plan for investigating causes of heterogeneity, justification for any deviations from the protocol.

3. Did the review authors **explain their selection of the study designs for inclusion in the review**? YES/NO. For YES, the review should satisfy one of the following: explanation for including only RCTs, or explanation for including only NRSI, or explanation for including both RCTs and NRSI.

4. **Did the review authors use a comprehensive literature search strategy**? YES, PARTIAL YES, NO. for PARTIAL YES must have all of the following: searched at least 2 databases (relevant to research question), provided key word and/or search strategy, justified publication restrictions (eg. Language). For YES should also have all of the following: searched the reference lists/biographies of included studies, searched trial/study registries, included/consulted content experts in the field, searched for grey literature where relevant, conducted search within 24 months of completion of the review.

5. Did the review authors perform **study selection in duplicate**? YES/NO. for YES, either ONE of the following: at least two reviewers independently agreed on selection of eligible studies and achieved consensus on which studies to include OR two reviewers selected a sample of eligible studies and achieved good agreement (at least 80 per cent) with the remainder selected by one reviewer.

6. Did the review authors perform **data extraction in duplicate**? YES/NO. For YES, either one of the following: at least two reviewers achieved consensus on which data to extract from included studies OR two reviewers extracted data from a sample of eligible studies and achieved good agreement (at least 80 per cent) with the remainder extracted by one reviewer.

7. Did the review authors provide **a list of excluded studies to justify the exclusions**? YES, PARTIAL YES, NO. FOR partial yes must provide a list of all potentially relevant studies that were read in full text form but excluded from the review. For YES must also have justified the exclusion from the review of each potentially relevant study.

8. Did the review authors **describe the included studies in adequate detail**? YES, PARTIAL YES, NO. For PARTIAL YES, must describe all of the following: populations, interventions, comparators, outcomes, research designs. For YES should also have all of the following: described populations in detail, described intervention and comparator in detail (including doses where relevant), described study setting, timeframe or follow-up.

9. Did the review authors use a **satisfactory technique for assessing the risk of bias (RoB) in individual studies** that were included in the review? For RCTs: YES, PARTIAL YES, NO, INCLUDES ONLY NRSI. For PARTIAL YES must have assessed RoB from unconcealed allocation and lack of blinding of patients and assessors when assessing outcomes (unnecessary for objective outcomes such as all cause mortality); for YES must also have assessed RoB from allocation sequence that was not truly random and selection of the reported result from among multiple measurements or analyses of a specified outcome. For NRSI (Non Randomized Studies of Intervention): YES, PARTIAL YES, NO, INCLUDES ONLY RCTs. For PARTIAL YES must have assessed RoB from confounding and from selection bias. For YES, must also have assessed methods used to ascertain exposures and outcomes, and selection of the reported results from among multiple measurements or analyses of a specified outcome.

10. Did the review authors report on the **sources of funding for the studies included in the review**? YES/NO. For YES: must have reported on the sources of funding for individual studies included in the review. Note: reporting that the reviewers looked for this information but it was not reported by study authors also qualifies

11. If meta-analysis was performed, did the review authors use **appropriate methods for statistical combination of results**? For RCTs: YES, NO, NO META-ANALYSIS. For YES: the authors justified combining the data in a meta-analysis and they used an appropriate weighted technique to combine study results and adjusted for heterogeneity if present and investigated the causes of heterogeneity. For NRSI: YES, NO, NO META-ANALYSIS CONDUCTED. For YES: the authors justified combining the data in a meta-analysis and they used an appropriate weighted technique to combine study results, adjusting for heterogeneity if present, and they statistically combined effects estimates from NRSI that were adjusted for confounding, rather than combining raw data, or justified combining raw data when adjusted effect estimates were not available, and they reported separate summary estimates for RCTs and NRSI separately when both were included in the review.

12. If meta-analysis was performed, did the review authors assess the **potential impact of RoB in individual studies on the results of the meta-analysis or other evidence synthesis?** YES, NO, NO META-ANALYSIS INCLUDED. For YES: included only low risk of bias RCTs or, if the pooled estimate was based on RCTs and/or NRSI at variable RoB, the authors performed analysis ton investigate possible impact of RoB on summary estimates of effect.

13. Did the review authors account **for RoB in individual studies** when interpreting/discussing the results of the review? YES/NO. for YES: included only low risk of bias RCTs or, if RCTs with moderate or high RoB, or NRSI were included, the review provided a discussion of the key impact of RoB on the results

14. Did the review authors **provide a satisfactory explanation for, and discussion of, any heterogeneity observed in the results of the review**? YES/NO. For Yes: there was no significant heterogeneity in the results OR if heterogeneity was present the authors performed an investigation of sources of any heterogeneity in the results and discussed the impact of this on the results of the review

15. If they performed quantitative synthesis **did the review authors carry out an adequate investigation of publication bias (small study bias) and** discuss its likely impact on the results of the review? YES, NO, NO META-ANALYSIS CONDUCTED. For YES: performed graphical statistical tests for publication bias and discussed the likelihood and magnitude of impact of publication bias

16. Did the review **authors report any potential sources of conflict of interest**, including any funding they received for conducting the review? YES/NO. For Yes: the authors reported no competing interests OR the authors described their funding sources and how they managed potential conflicts of interest.

Rating overall confidence in the results of the review:

HIGH: no on one non-critical weakness: the systematic review provides an accurate and comprehensive summary of the results of the available studies that address the question of interest

MODERATE: more than one non critical weakness (multiple non-critical weaknesses may diminish confidence in the review and it may be appropriate to move the overall appraisal down from moderate to low confidence): the systematic review has more than one weakness but no critical flaws. It may provide an accurate summary of the results of the available studies that were included in the review

LOW: one critical flaw with or without non-critical weaknesses: the review has a critical flaw and may not provide an accurate and comprehensive summary of the available studies that address the question of interest

CRITICALLY LOW: more than one critical flaw with or without non-critical weaknesses: the review has more than one critical flaw and should not be relied on to provide an accurate and comprehensive summary of the available studies.
